# Quantifying Restoration Offsets at a Nuclear Power Plant in Canada

**DOI:** 10.1007/s00267-019-01214-2

**Published:** 2019-10-19

**Authors:** Lawrence W. Barnthouse, Cherie-Lee Fietsch, David Snider

**Affiliations:** 1 LWB Environmental Services, Inc., 1620 New London Rd., Hamilton, OH 45013 USA; 2 Bruce Power LP, 177 Tie Rd., Tiverton, ON N0G2T0 USA

**Keywords:** Impingement, Entrainment, Equivalent adult model, Habitat productivity index, Canadian *Fisheries Act*

## Abstract

In Canada, the *Fisheries Act* requires all water takers to avoid, mitigate and offset fish losses. To satisfy the act’s requirements, operators of power plants are required to undertake habitat restoration projects to compensate for fish impinged and entrained at cooling water intake structures. Scaling the quantity of restoration needed, and measuring whether adequate compensation has been achieved, requires a metric that expresses the losses and gains in comparable units. Development of such a metric is especially difficult in the case of power plants, because the losses often consist of a mix of species and life stages that are very different from those produced by technically feasible restoration projects. This paper documents the method that has been developed for quantifying offsets for impingement and entrainment at the Bruce Generating Stations on the eastern shore of Lake Huron, and demonstrates how the method is being used to estimate the offset to be provided by removal of a dam on the nearby Saugeen River.

## Introduction

The Canadian *Fisheries Act* was amended in 2012 to establish Fisheries Protection Provisions that protect ongoing fishery productivity. The amended act requires proponents of existing or proposed works, undertaking, or activities, including operators of power plants, to avoid or mitigate “serious harm to fish,” which includes direct fish mortality, and destruction or permanent alteration of fish habitat (Fisheries and Oceans Canada 2013a). If “serious harm to fish” is likely to occur despite the application of avoidance and mitigation measures, then owners or operators are required to undertake offsetting measures to counterbalance that harm.

The goal of Fisheries Protection Policy in Canada is to “provide for the sustainability and ongoing productivity of commercial, recreational and Aboriginal fisheries” (Fisheries and Oceans Canada 2013b). One way to support this policy is to ensure that development activities produce no net loss of the productive capacity of Canadian fish habitat (Minns et al. 2011). To comply with the no net loss requirement, project developers must first identify the types of impacts on productive capacity caused by the project and then develop metrics for quantifying and balancing the losses and gains.

The great majority of projects subject to the requirements of the *Fisheries Act* involve construction activities that physically alter fish habitat, even though they may cause little or no direct mortality to fish. These habitat losses can be offset by a variety of habitat restoration or habitat creation projects, and a variety of resources are available to aid project developers (e.g., de Kirkhove et al. 2008; Minns et al. 2011; Fisheries and Oceans Canada 2013b, 2014; Abdel-Fatah et al. 2017).

For example, Minns et al. (2011) reviewed the available approaches to assessing the reduction in productive capacity resulting from development projects and determining the amount of compensatory restoration required to offset the project-related reductions. Most of these approaches utilize measurements of surrogate habitat variables such as depth, substrate type, and cover type that are believed to be related to fish production rather than actual measurements of production. To facilitate implementation of habitat-based methods, a web-based tool termed the HEAT model (Abdel-Fatah et al. 2017) is being developed to aid in quantifying the suitability of different types of habitat for Canadian fish species. Given information on the physical characteristics of the fish habitat present at a project site, the fish species expected to be present, the area potentially affected by the project, and the types of habitat changes expected, the HEAT model calculates a surrogate for productive capacity termed the “weighted useable area” (WUA) for the project area both before and after development. The difference between these two values is the net gain or loss of productive capacity due to the project. The project developer must then restore or create habitat with the same WUA value as the net loss from development.

**Fig. 1:**
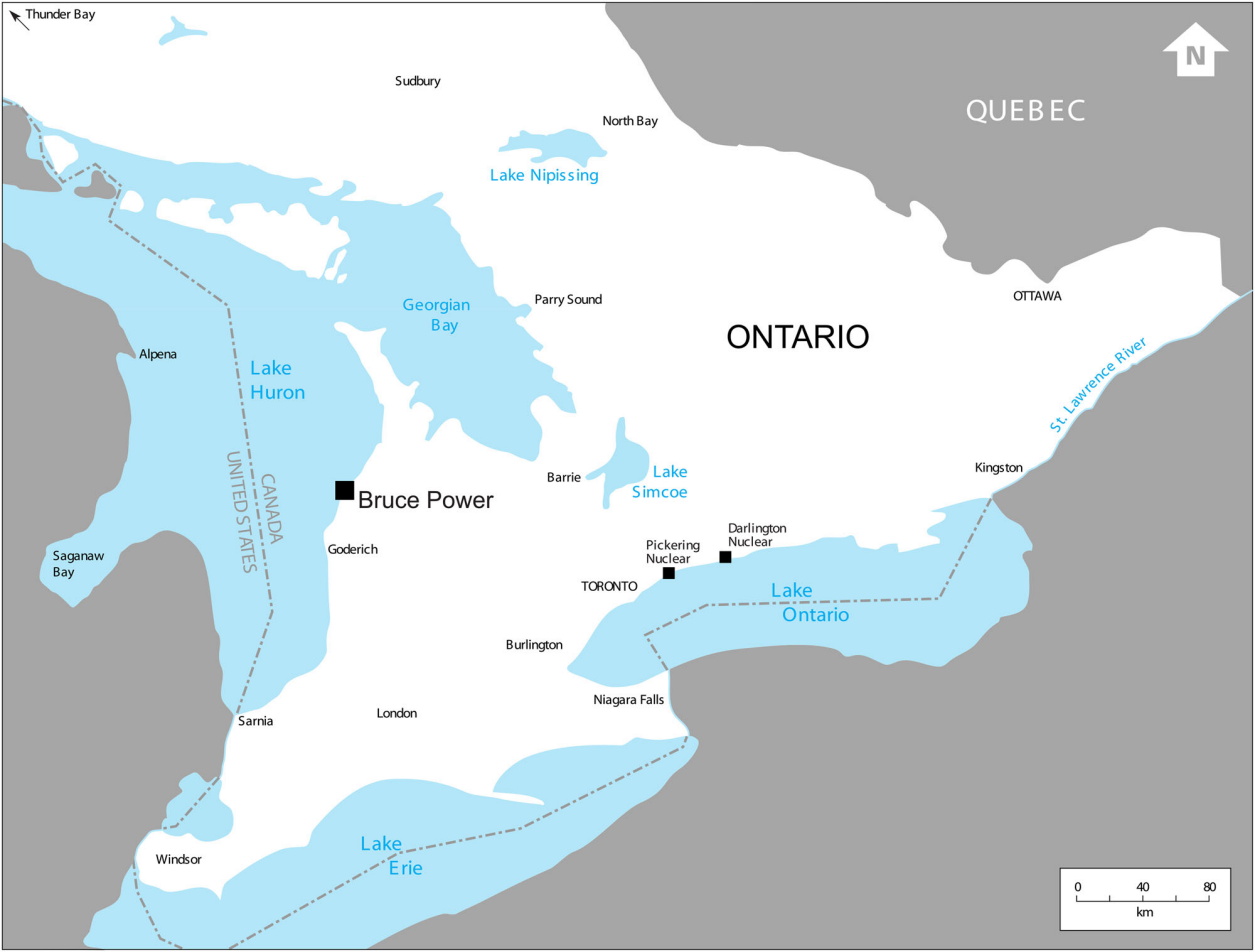
Location of Bruce Station on the eastern shore of Lake Huron

The methods that have been used to quantify offset requirements for facility construction projects have, at best, limited applicability to operating power plants such as the Bruce Generating Stations (Fig. 1). The “serious harm” caused by the operation of existing power plants is due to direct mortality: impingement (entrapment) of fish on the traveling screens that prevent debris from being drawn into the plant’s cooling water system, and the drawing through the cooling water system (entrainment) of early life stages of fish that cannot be retained on the traveling screens. Impingement and entrainment at power plants has been a major topic of scientific and regulatory concern at least since the 1970s (Fisheries and Oceans Canada 1991; USEPA 1977; USEPA 2014). No physical habitat is altered, therefore, the habitat-based methods discussed by Minns et al. (2011) and implemented in the HEAT tool (Abdel-Fatah et al. 2017) are not applicable. However, even using the most advanced technologies for reducing impingement and entrainment, some losses of fish are inevitable and are subject to the offset requirements of the *Fisheries Act*.

A significant complicating factor is that the species impinged and entrained may not be the same as the species produced through habitat restoration or other types of offsets. The fish species impinged and entrained at the Bruce Generating Stations include pelagic species such as Lake Whitefish (*Coregonus clupeaformis*) and Lake Trout (*Salvelinus namaycush*), benthic species such Channel Catfish (*Ictalurus punctatus*) and suckers, and nearshore species such as Smallmouth Bass (*Micropterus dolomieu*) and Yellow Perch (*Perca flavescens*). Opportunities for increasing the production of these species are limited because of both the high diversity of habitat types involved and the fact that fish habitat in eastern Lake Huron is relatively unimpaired. In contrast, opportunities to increase fish production are available in local tributaries to Lake Huron, which have been historically impaired by dam construction, channelization, sediment deposition, and other environmental stressors. “Out of kind” offsets produced by restoration of these habitats could significantly enhance fishery resources in the Lake Huron watershed. Out-of-kind offsets are accepted alternatives when in-kind offsets are not possible, or do not fully meet the offsetting needs (Fisheries and Oceans Canada 2013c).

In the long term, restoration of freshwater habitats can provide more than adequate replacement of fish lost due to impingement and entrainment, although the replacements consist of species and life stages different from those that are impinged and entrained. In the short term, Bruce Power intends to provide interim offsets in the form of stocking of Lake Huron with a fish species that supports restoration of ecological function. Metrics applicable to both of these kinds of offsets are needed.

These metrics must be expressed in units applicable both to entrainment and impingement counts and to data obtained from field surveys in systems expected to provide restoration offsets. The remainder of this paper documents the development and demonstration of metrics applicable to scaling restoration offsets and measuring success for the Bruce Generating Stations.

## The Equivalent Adult (EA) Approach

Station-related losses of fish consist of a mix of life stages and age groups, members of which have different probabilities of surviving to contribute to future population growth and harvest. Combining losses of multiple ages and life stages into a common metric is necessary not only for scaling restoration projects, but also for calculating economic benefits of mitigating measures intended to reduce those losses (USEPA 2002, 2014). Benefits analyses have been performed in the United States for many years and are required for compliance with the U.S. Clean Water Act’s §316(b) rule (USEPA 2014). The EA model (Horst 1975) is the most widely-used method for accomplishing this task. Given estimates of stage- and age-specific mortality rates, the EA model converts estimates of numbers of fish impinged or entrained at multiple ages and life stages to numbers that would have survived to some arbitrary common age, termed the “age of equivalence” (EPRI 2004). In analyses performed to support §316(b) rule-making, USEPA had consistently adopted an age of 1 year as the age of equivalence (USEPA 2002, 2014). USEPA’s practice is followed in most benefits analyses performed by permit applicants. The losses are expressed as numbers of 1-year-old equivalent fish, or, using the average weight of a 1-year-old fish, the age-1 equivalent biomass lost. Because the EA model is so widely used, estimates of the required mortality rates and age-specific weights are readily available in published literature.

In principle, the EA model can be used to calculate offset requirements that satisfy the requirements of the Canadian *Fisheries Act*. For this purpose, the most straightforward approach would be to express both the losses from impingement and entrainment and the gains from stocking or restoration in terms of age-1 equivalents. Because the losses and gains are likely to be from different species, total age-1 equivalent biomass over all impinged and entrained species would be the most appropriate unit for comparison.

There are two problems with using the EA model to scale offset projects. First, as discussed below, the EA model can produce unrealistically inflated estimates of age-1 equivalent losses when applied to fish impinged at ages older than 1 year. Second, although easily applied to counts of numbers of fish impinged and entrained, the EA model is difficult to apply to field data from typical fish community surveys, especially if the project is an “out-of-kind” offset and the biomass monitoring is carried out in a different ecosystem where mortality rates and age-specific weights are not readily known.

### Inflated Estimates of Age-1 Equivalent Losses

Although the EA model has been used in impingement and entrainment impact assessments for 40 years, it has a significant flaw that has been largely unrecognized. This flaw is important only in situations in which some impinged fish are older than the chosen age of equivalence. Such situations are uncommon, because the majority of losses of fish at most power plants consist of eggs, larvae, and young-of-the-year fish. However, at some plants, including the Bruce Generating Stations, fish that are much older than age-1 are commonly impinged. In these cases, application of the EA model can lead to unrealistically inflated estimates of age-1 equivalent losses.

Assuming that age-1 is chosen as the age of equivalence, the EA model is expressed as:

1 $${\rm{NEQ}}_{1} = \mathop {\sum}\limits_{i = 1}^{n} {S_{i,1}N_{i}},$$

where *NEQ*_*1*_ = number of age-1 equivalent fish, *N*_*i*_ = number of fish impinged or entrained at age or stage *i*, *S*_*i,1*_*=* fraction of fish expected to survive from age or stage *i* to age-1.

As long as age or stage *i* is less than 1, losses are being projected forward to the number that would have survived to become 1-year-old fish, had they not been impinged or entrained. The problem arises when age *i* is greater than 1. The simplest approach would be simply to run the standard EA model backwards, projecting the number of age-1 fish required to produce each older fish. Mathematically, this involves dividing the number of fish impinged at age *x* (>1) by the survival rate of fish from age-1 to age *x*:

2 $${\rm{NEQ}}_{1} = \frac{{N_{x}}}{{S_{1,x}}},$$

where *S*_*1,x*_ = fraction of fish expected to survive from age-1 to age *x*

This calculation, although mathematically logical, is biologically unrealistic. The reason is that projecting impinged or entrained fish forward to older ages is fundamentally different from projecting backwards to earlier ages. Impingement and entrainment eliminate all future growth, reproduction, and harvest for those fish impinged or entrained. Projecting these losses forward from earlier ages to age 1 provides a common metric from which future losses of harvest, production, or reproduction can be calculated. However, when projecting backward to age-1 from older ages, the expanded numbers of fish consist of fish that were harvested or died of natural causes between age 1 and age *x* and were never impinged. All of these fish performed their natural ecological functions for their entire lifespans. They should not be counted as station-related losses, and do not provide a reasonable basis for calculating offset requirements.

Impingement losses at the Bruce Generating Stations provide an illuminating illustration of this problem. Lake Whitefish as old as age-12 are impinged. Life history parameters for Lake Whitefish used to apply the EA model to these fish are listed in Table 1. The estimated total annual instantaneous mortality is 0.45 at ages 1–3, 0.65 at age 4, and 0.9 for ages 5 and older^1^. The higher mortality rate for age-4 and older fish is due to harvesting. These values imply a probability of survival of 0.025% from age-1 to age-12. In EA modeling, fish are conventionally assumed to be impinged or entrained randomly within the stage or age of loss. To account for this pattern, a survival rate adjustment is applied to the stage or age at which entrainment or impingement occurs (USEPA 2002; EPRI 2004):

3 $$S_{x}^ {\ast} = 2S_{x}e^{ - \ln \left( {1 + S_{x}} \right)}.$$

**Table 1:** Life history parameters for lake whitefish

Age	Annual total mortality^a^	Annual survival	Age-1 to Age × survival	% Mature^b^	Annual fecundity^c^
1	0.45	0.638	1	0%	0
2	0.45	0.638	0.779	0%	0
3	0.45	0.638	0.497	0%	0
4	0.65	0.522	0.317	50%	27,858
5	0.90	0.407	0.165	100%	35,033
6	0.90	0.407	0.067	100%	40,322
7	0.90	0.407	0.027	100%	44,043
8	0.90	0.407	0.011	100%	46,587
9	0.90	0.407	0.005	100%	48,296
10	0.90	0.407	0.002	100%	49,431
11	0.90	0.407	0.001	100%	50,180
12	0.90	0.407	0.000	100%	50,672
13	0.90	0.407	0.000	100%	50,994
14	0.90	0.407	0.000	100%	51,204

^a^Age-specific total mortality rates are from Muir et al. (2008) and Ebener et al. (2010). Lake whitefish were assumed to first become vulnerable to harvesting at age 4, and to be fully recruited to the fishery by age 5. Natural mortality and fishing mortality were assumed to contribute equally to total mortality of fish aged 5 years and older.

^b^From Wang et al. (2008)

^c^From Muir et al. (2014)

Assuming that impingement losses of age-12 fish are randomly distributed throughout the year, the average impinged fish would have survived 57.8% of the interval between its 12th and 13th birthdays. Accounting for this additional mortality, 0.014% of age-1 Lake Whitefish would be expected to survive to be impinged at some point between ages 12 and 13. Using the unadjusted EA model, the number of age-1 equivalent 12-year-old fish would be calculated by dividing the number of impinged fish by the probability of surviving from age-1 to age-12. Using this procedure, each of the 28 12-year-old Lake Whitefish impinged at Bruce Generating Stations in 2013 would expand to ~7000 age-1 equivalents. Together, these 28 fish would expand to 195,000 age-1 equivalents. Assuming that a 1-year-old Lake Whitefish weighs 75 g, 195,000 age-1 equivalents would correspond to 14,569 kg of age-1 Lake Whitefish. As discussed above, expressing fish older than 1-year as age-1 equivalents over-inflates the actual losses because the vast majority of these fish died of natural causes between age-1 and age-12. All but 12 of the 195,000 fish were never exposed to the plant’s intake structure, and should not be counted as station-related losses for which offsets are required.

The fallacy inherent in using Eq. 2 to expand numbers of 12-year-old fish to numbers of age-1 equivalents can be further demonstrated using Rago’s (1984) Production Foregone model. This model is commonly used to estimate the loss in fish production available to predators and fishermen due to impingement and entrainment.

The production foregone at any given life stage or age is calculated by integrating the instantaneous rates of growth and mortality over that stage or age:

4 $$P_i = \frac{{G_iN_i\overline W _i\left( {\exp \left( {G_i - Z_i} \right) - 1} \right)}}{{G_i - Z_i}},$$

where *P*_*i*_ = production foregone for a specific age or life stage *i*. *G*_*i*_ = instantaneous growth rate for individuals of age or life stage *i*. *Z*_*i*_ = instantaneous total mortality rate for individuals of age or life stage *i*. $\overline W _i$ = average weight of individuals at the beginning of age or life stage *i.*

Equation 4 applies only to the age or stage at which a particular fish or group of fishes is entrained or impinged. To account for all of the production foregone over the expected lifetimes of these fish had they not been entrained or impinged, Eq. 4 has to be applied to all future ages through the full life span of the species. However, the production at each future age or stage *j* must be adjusted to account for mortality occurring between stage or ages i and *j*:

5 $$P_{i,j} = P_jS_{i,j},$$

where *S*_*i,j*_ = fraction of fish expected to survive from age or stage *i* to stage or age *j*

The total lifetime production foregone due to entrainment or impingement is then calculated by summing the age or stage-specific values over all future ages:

6 $$P_T = \mathop {\sum}\limits_{j = i}^{A_{\rm{max}}} {P_{i,j}}$$

To account for all of the biomass lost to the ecosystem due to entrainment and impingement, the weight of the entrained or impinged fish are then added to the production foregone.

Table 2 illustrates the application of the Production Foregone model to 12-year-old Lake Whitefish. By age-12, Lake Whitefish have grown to nearly their maximum length and weight, so that there is little potential for future production. As shown in Table 2, the biomass lost to the ecosystem due to impingement of these fish is only 1.98 kg per fish, nearly all of which is accounted for by the weight of the fish at impingement. The 28 impinged 12-year-olds account for only a 55 kg reduction in biomass available to the Lake Huron ecosystem.

**Table 2:** Calculation of lifetime production foregone (g) for age-12 Lake Whitefish (PF) using Rago’s (1984) model

Age	Weight (g)	*G*	*Z*	Age-12 to age *j* survival	stage-specific PF per fish	Cumulative PF per age-12
12	1969.11	0.006335	0.9	1	8.25E + 00	8.25E + 00
13	1981.62	0.004118	0.9	0.40657	5.39E + 00	1.04E + 01
14	1989.80	0	0.9	0.165299	0.00E + 00	1.04E + 01
Total biomass lost to the ecosystem						1.98E + 03

Table 3 illustrates the application of the Production Foregone model to 1-year-old Lake Whitefish. A 1-year-old fish has a substantial capacity for future production, even accounting for mortality at future ages. As Table 3 shows, the expected lifetime production of each age-1 Lake Whitefish is estimated to be 575 g, 500 of which is being attributed to future growth. The 195,000 age-1 equivalents calculated using Eq. 2 would be expected to produce 128,000 kg of biomass over their lifetimes, or *more than 2300 times the biomass actually lost due to impingement of 28 12-year-old fish*. This inconsistency further demonstrates that Eq. 3 cannot be validly used to establish offset requirements for fish impinged at ages older than 1 year.

**Table 3:** Calculation of lifetime production foregone for age-1 Lake Whitefish (PF) using Rago’s (1984) model

Age	Weight (g)	*G*	*Z*	Age-specific PF per fish	Age-1 to age *j* survival	Cumulative PF per age-1 fish (g)
1	75.81	1.559597	0.45	2.17E + 02	1	2.17E + 02
2	360.61	0.710836	0.45	2.93E + 02	0.637628	4.03E + 02
3	734.08	0.388475	0.45	2.77E + 02	0.40657	5.16E + 02
4	1082.58	0.229146	0.65	2.02E + 02	0.212248	5.59E + 02
5	1361.37	0.140619	0.9	1.34E + 02	0.086294	5.70E + 02
6	1566.91	0.088262	0.9	9.47E + 01	0.035084	5.74E + 02
7	1711.50	0.056155	0.9	6.49E + 01	0.014264	5.75E + 02
8	1810.36	0.036028	0.9	4.37E + 01	0.005799	5.75E + 02
9	1876.77	0.023238	0.9	2.90E + 01	0.002358	5.75E + 02
10	1920.89	0.015039	0.9	1.92E + 01	0.000959	5.75E + 02
11	1950.00	0.009754	0.9	1.26E + 01	0.00039	5.75E + 02
12	1969.11	0.006335	0.9	8.25E + 00	0.000158	5.75E + 02
13	1981.62	0.004118	0.9	5.39E + 00	6.44E-05	5.75E + 02
14	1989.80	0	0.9	0.00E + 00	2.62E-05	5.75E + 02
Total biomass lost to the ecosystem						6.51E + 02

The Appendix documents a method for correcting the EA model, based on the well-established theory of reproductive value. The corrected model weights each impinged fish according to the remaining potential egg production of a fish at the age of impingement, compared to the potential lifetime egg production of a 1-year-old fish. Using the corrected model, the 28 12-year-old Lake Whitefish have the same reproductive potential as 363 1-year-old fish. Stated another way, removing 28 12-year-old lake whitefish from the total Lake Whitefish population has the same impact on the ability of the population to sustain itself as removing 363 1-year-olds. Equivalently, producing 363 new 1-year olds would be sufficient to replace the reproductive capacity of the 28 12-year-old fish. As demonstrated in the Appendix, the adjusted model also correctly accounts for the relative reproductive value of eggs and larvae as compared to 1-year-old fish.

Stocking may be used as an interim measure for power plants which continually impinge and entrain fish and require a short-term measure to offset losses while longer term offsets are in development. As corrected using the reproductive value adjustment, the EA model provides an internally consistent metric for calculating the biomass of 1-year-old fish that may be stocked annually to offset I&E losses. This can be done simply by extrapolating losses of all fish species to age-1 equivalent biomass using the appropriate life history parameters and summing the species-specific values to obtain the total age-1 equivalent I&E losses over all species.

### Difficulties in applying the EA model to field survey data

If the requirements of the *Fisheries Act* could be met solely by stocking of hatchery-produced fish, then the corrected EA model would be sufficient for calculating the necessary offset. However, guiding principles of the Fisheries Protection Policy require that offsetting measures must generate self-sustaining benefits over the long-term (Fisheries and Oceans Canada 2013c), and restoration or enhancement of fish habitat is preferred over fish stocking.

Restoration projects available for offsetting impingement and entrainment at Bruce Generating Stations involve dam removals and in-stream habitat enhancements, both of which can be expected to increase fish production. Well-established methods, typically based on electrofishing, are available for estimating the changes in fish populations and communities resulting from restoration activities. However, there is a significant disconnect between the data provided by electrofishing surveys and the data provided by monitoring impingement and entrainment at power plants. Impingement and entrainment sampling can be conducted as frequently as necessary to quantify the losses to any desired degree of precision. At some plants, entrainment sampling is conducted weekly or even several times per week. Impingement sampling at some plants, including Bruce Power, is conducted daily. Estimates of stage-and age-specific mortality rates for most commonly impinged and entrained species are available and can be used to convert the losses to age-1 equivalents.

In contrast, field surveys provide a snapshot of fish population and community structure at a specific location and time. Surveys like those described in this paper are complex and costly to conduct and cannot reasonably be performed more than a few times per year. Although the lengths and weights of the fish collected are routinely tabulated, ages are often unknown. To apply the EA model, it would be necessary to identify, for each fish collected, its age in months or weeks relative to an age of 1 year, and then to project either forwards or backwards to age-1. Age–length relationships for the small fish species that dominate production in rivers and streams are not readily available. Although it may be possible in principle to perform these calculations, no established methods for doing so are available in agency guidance or peer-reviewed literature. Moreover, only fish large enough to be captured by electrofishing can be collected; fish eggs, larvae, and early juveniles typically are not collected in elecrofishing surveys. No method for quantitative sampling of fish eggs and larvae in streams exists. These life stages are difficult to sample in stream environments because eggs are often attached to solid substrates and larvae are restricted to nests or other microhabitats where current velocities are low enough to prevent washout. Without quantitative field sampling, it is not possible to compare ichthyoplankton data collected in the field to ichthyoplankton data collected by entrainment studies.

## Calculating Long Term Offsetting Benefits: the Habitat Productivity Approach

Comparing losses due to I&E to gains from habitat restoration requires a metric that applies equally to both types of fisheries data. Minns et al. (2011) reviewed the available approaches to assessing the reduction in fish productive capacity resulting from development projects and determining the amount of compensatory restoration required to offset the project-related reductions. As discussed above, the most widely-used approaches are based on measurements of physical habitat characteristics and are not applicable to I&E at power plants.

However, another approach discussed by Minns et al. (2011), termed the habitat productivity index (HPI) approach, utilizes species-specific production–biomass (*P*/*B*) ratios and produces outputs expressed as annual fish *production* rather than productive capacity as a habitat metric. Production is a metric that can be applied to I&E data as well as to field-collected fishery data, therefore it can be used to calculate offset requirements for the Bruce Generating Stations.

The scientific literature defines the “production” of a fish population as “…the total elaboration of new tissue in a time period of interest by a species-population“ (Chapman 1978). Production over any interval time is equal to the mean biomass of the population during that interval multiplied by the growth rates of the fish during this period (Ricker 1946). Production in fish populations is difficult to measure in the field, however, research has shown that the relationship between production and biomass, generally expressed as the production to biomass (P/B) ratio, is inversely related to fish size and varies over a range of ~0.2 to 5 (Randall and Minns 2000).

For any fish population *i*, total annual production can be approximated as:

7 $$P_i = B_i\left( {P{\mathrm{/}}B} \right)_i,$$

where *P*_*i*_ is total production, B_*i*_ is the average annual population biomass, and (*P*/*B*)_*i*_ is a species-specific production to biomass ratio.

Species-specific *P*/*B* ratios are inversely related to body size, with larger species having lower (P/B) ratios than smaller species. Randall and Minns (2000) found that (*P*/*B*) ratios for Canadian freshwater fish species could be estimated from species-specific weights-at-maturity using the following equation:

8 $$\left( {P{\mathrm{/}}B} \right)_i = 2.64 \times {\rm{WMAT}}_i^{ - 0.35},$$

where WMAT_*i*_ is the average weight (g) at maturity of fish species *i*.

Subsequently, Randall and Minns (2002) and Randall et al. (2017) found that similar results could be obtained by using estimates of average fish weight in grams (*W*_*i*_) from electrofishing data rather than weight-at-maturity to calculate *P*/*B* ratios.

For the community of fish inhabiting a particular habitat, a habitat productivity index (HPI) can be estimated simply by summing the annual production of all of the fish species present:

9 $$HPI = {\sum} {B_i\left( {\frac{P}{B}} \right)_i}.$$

In applying the HPI approach to field data, biomass values (*B*_*i*_) are often expressed as biomass density, e.g., biomass per unit area. To estimate the total fish biomass present in a community, biomass density values are multiplied by the total area of the habitat being evaluated. The HPI approach, like the classical approaches to fish production calculation documented by Ricker (1946) and Chapman (1978), assumes that the fish collected are resident fish that are contributing to community fish production. The presence of migratory fish that are simply passing through a site could bias the production estimates. Hence, care must be taken to perform surveys outside the migration season, or to eliminate migratory fish from the production calculations.

Unlike the EA approach, the HPI approach can easily be applied to field data collected in rivers or streams identified as restoration sites. Field studies must be performed before and after restoration; the difference between before and after HPI values is a measure of the increase or decrease in productivity due to restoration.

With a slight modification, the HPI approach can also be applied to I&E data collected at power plants. Provided that the weights of impinged fish are measured during in-plant monitoring or can be calculated from known length–weight relationships, the HPI model can be directly applied to impingement data. In effect, the impingement collection is treated as if it were an electrofishing sample. The HPI value calculated from the impingement data is expressed in exactly the same units as an HPI value calculated from a field sample, and therefore can be used to scale the production required from restoration projects.

Application of the HPI approach to entrainment data requires an adjustment to the model. Electrofishing surveys do not provide estimates of the abundance or biomass of early life stages of fish present in streams or rivers, so the production of these life stages is not included in field-derived HPI estimates. However, it is still necessary to account for the contribution of eggs and larvae to the lost production due to I&E. Eggs and larvae entrained at the Station have extremely low weights and accurate measurements are not possible in the field. It is much more effective to simply project them to age-1 equivalents. When multiplied by the average weight of a 1-year-old fish, these estimates are directly comparable to estimated weights of impinged age-1 fish. As shown in the Appendix, projections of eggs and larvae to age 1 are identical using both the original and the adjusted age-1 equivalent models. Once the entrainment losses have been converted to age-1 equivalents, they are added to the age-1 impingement biomass used in the HPI calculations.

## Application of EA and HPI models to I&E data for the Bruce Generating Stations: Lake Whitefish Example

An estimated 100,000 Lake Whitefish larvae were entrained and 73 Lake Whitefish juveniles and adults were impinged at the Bruce Generating Stations in 2013 (Table 4). Applying the corrected EA model documented in the Appendix, these losses equate to 3947 individuals of age-1 equivalent fish. Approximately 90% of these age-1 equivalents result from entrainment of larvae. Using age–length and length–weight data provided for Lake Huron by the Ontario Ministry of Natural Resources, the estimated average weight of an age-1 Lake Whitefish is 75 g. Using this value, the total age-1 equivalent biomass of Lake Whitefish lost at the Bruce Generating Stations in 2013 is estimated to be 295.1 kg.

**Table 4:** Application of equivalent adult (EA) and habitat productivity index (HPI) models to lake whitefish impingement and entrainment data for Bruce Station^a^, 2013

Life stage or age (years)	Number impinged or entrained	Annual loss as age-1 equivalents^b^	Annual age-1 equivalent biomass loss (kg)	Age-specific weight (g)	Total biomass (g)
Larvae	100,000	3433	256.6		
Juveniles	12	1	0.1		
1	9	12	0.9	75	258,422^c^
2	10	25	1.8	361	3606
3	4	15	1.2	734	2936
4	4	27	2.1	1083	4330
5	2	21	1.6	1361	2723
6	2	24	1.8	1567	3134
7	2	25	1.9	1711	3423
8	0	0	0.0	1810	0
9	0	0	0.0	1877	0
10	0	0	0.0	1921	0
11	0	0	0.0	1950	0
12	28	363	27.2	1969	55,135
13	0	0	0.0	1982	0
14	0	0	0.0	1990	0
Total		3947	295.1		333,709
Average individual biomass (g)					95
*P*/*B* ratio					0.54
HPI (kg)					179.3

^a^Bruce A and Bruce B combined

^b^Calculated according to corrected EA model, as documented in Appendix

^c^Includes larvae and juveniles as age-1 equivalents

For the purpose of offsetting as described in the Fisheries Productivity Investment Policy (Fisheries and Oceans Canada 2013c) a proponent may choose to stock hatchery fish as a short-term strategy to immediately offset all fish losses. The adjusted EA model is a convenient and accurate way to calculate the amount of hatchery fish needed to fully offset impingement and entrainment losses because the age of equivalence can be matched to the age of the stocked fish. Using the Lake Whitefish example provided above, 295.1 kg of 1-year-old hatchery fish could sufficiently offset the losses of Lake Whitefish incurred in 2013.

To quantify long-term offsets achieved through habitat restoration we apply the HPI methodology to both station losses and ecosystem gains in production. Table 4 demonstrates application of the HPI model to I&E data for Lake Whitefish impinged and entrained at the Bruce Generating Stations in 2013. This calculation combines the actual weights of age-1 and older fish with the estimated age-1 equivalent weights of larvae and juveniles (333,709 g, Table 4). The average weight of all fish was calculated to be 95 g, corresponding to a P/B ratio of 0.54 (Eq. 4). Combining this P/B ratio with a total biomass of 333.8 kg, the loss in production of Lake Whitefish due to I&E is calculated as 179.3 kg/year. For the purpose of long-term offsetting, restoration of fish habitat sufficient to produce 179.3 kg/year of new fish biomass would be required to offset the loss of Lake Whitefish production due to I&E.

### Scaling Potential Offset Project(s)

Quantifying the potential value of an out-of-kind offset project in numerical terms is difficult because defensible methodologies accepted by the scientific community are scarce. Guidance documentation provided by government agencies is very basic and the body of peer-reviewed scientific literature available is small (Clarke and Bradford 2014). This inability to quantifiably link restoration activities to fisheries production is a knowledge gap identified in DFO guidance documents (Barrell et al. 2014). Accurate equivalency models cannot be created without support from empirical evidence, and the body of literature reporting fish biomass before and after restoration work is very small. Nonetheless, operators and project applicants in Canada are required to provide numerical estimates of the amount of productive capacity that will be generated from offset projects to receive authorization under the *Fisheries Act*.

Although the required offset should in principle be expressed in terms of annual production (kg/year), most studies of impacts of restoration on fish communities provide estimates of changes in standing fish biomass (kg). However, published literature (Neves and Pardue 1983; Portt et al. 1986; Randall et al. 2017) indicates that P/B ratios for fish communities vary over a range of approximately 1–2. Hence, for the purpose of predicting offsets, standing biomass estimates obtained from published literature are reasonable surrogates for annual fish community production.

Reports of fish biomass measured in Southern Ontario rivers and streams are available in the published literature. Bowlby and Roff (1986) reported the mean biomass of trout species (salmonids) in six Southern Ontario streams to be 3.02 g/m^2^. Randall et al. (2014) compiled biomass estimates for the entire fish community (34 fish species including salmonids) at 38 locations across 8 Southern Ontario streams (Rouge River, Lynde Creek, Humber River, 14 Mile Creek, Duffins Creek, Morningside Creek, Silver Creek, and 16 Mile Creek). The reported median biomass of the entire fish community at these locations was 9.68 g/m^2^. Because these surveys were conducted in impacted rural and urban watersheds, the values derived from these studies are a good representation of the initial biomass conditions at sites available for restoration to offset I&E losses at the Bruce Generating Stations.

Removal of dams that restrict fish movement could provide the required offset, depending on the increase in production that results and on the size of the area within which the increase occurs. However, there are relatively few studies that quantify fish abundance before and after dam removals. Kornis et al. (2015) characterized the fish community present in Big Spring Creek, WI, before and after removal of a dam. Mean total fish biomass upstream from the dam site increased from 23.7–43.1 g/m^2^ following dam removal, in large part because of increased White Sucker (*Catastomus commersoni*) and Yellow Perch biomass post-removal. However, mean total downstream fish biomass declined from 45.7–21.0 g/m^2^ following dam removal. This decline reflected greatly decreased abundance of Bluegill (*Lepomis macrochirus*), which may have been affected by reduced water temperatures and increased suspended sediment loading.

Hogg et al. (2015) documented changes in the fish community of Sedgeunkedunk Stream, a small tributary to the Penobscot River, ME following removal of a dam that prevented upstream movement of anadromous Atlantic Salmon (*Salmo salar*), Alewife (*Alosa pseudoharengus*), and Sea Lamprey (*Petromyzon marinus*). The authors found that dam removal significantly enhanced the fish assemblage in Sedgeunkedunk Stream, including increased abundance of both resident and anadromous species. Tabular results were not provided by the authors, but examination of Fig. 3 of their paper indicates that 2 years following removal of the dam biomass density at the three study sites above the dam site was stabilizing at 2–12 g/m^2^ higher than at reference sites below the dam.

Table 5 summarizes the results of these two studies and documents an estimate of the relative change in fish biomass per unit area that was achieved through dam removal.

**Table 5:** Published values of fish biomass in riverine systems before and after barrier removal

Remediation Category	Species	Location	*B*_initial_ (g/m^2^)	*B*_final_ (g/m^2^)	Δ*B*	Ref
Barrier removal (e.g., dam removal)	Community	Sedgeunkedunk Stream, Maine, downstream	5	4	−0.2	Hogg et al. 2015
		Sedgeunkedunk Stream, Maine, upstream	2	11	4.5	
		Net ΔB (upstream + downstream)			4.3	
		Big Spring Creek, Wisconsin, downstream	45.7	21.0	−0.54	Kornis et al. 2015
		Big Spring Creek, Wisconsin, upstream	23.7	43.1	0.82	
		Net ΔB (upstream + downstream)			0.28	
	Average Net ΔB (upstream + downstream)				2.29	

The change in fish biomass (Δ*B*) before and after remediation can be expressed as a relative change, according to Eq. 10:

10 $$\Delta {B = }\frac{{{B}_{{\mathrm{final}}} - {B}_{{\mathrm{initial}}}}}{{{B}_{{\mathrm{initial}}}}},$$

where, *B*_final_ and *B*_initial_ are the final biomass and initial biomass, respectively of fish in a riverine system. The Δ*B* is a unitless factor by which the biomass increases or decreases, and can be expressed as a percent relative change (%) by multiplying Δ*B* by a factor of 100. Expressing this as a relative change is useful for comparing across study sites that have different initial standing biomass. The terms *B*_final_ and *B*_initial_ are the standing biomass present in an ecosystem at any given time and are expressed in units of mass per unit area (e.g., g/m^2^).

It is important to remember that long-term monitoring was not completed in either Sedgeunkedunk Stream or Big Spring Creek. Instead, these streams were monitored for 2–3 years prior to dam removal and for only 3 years afterward. Immediately following dam removal, downstream habitat can be temporarily degraded due to releases of sediment previously trapped behind the dam. In reality, several additional years beyond the 3-year monitoring periods might be required to stabilize the fish communities above and below the former dam site. Hence the Δ*B* value developed in Table 5 is best regarded as a preliminary value for project planning purposes, to be used only to provide an approximate estimate of the size of the restored area that might be required to offset I&E losses.

Given the estimates of Δ*B* compiled in Table 5, the mass (*m*) in kilograms of fish produced annually by a restoration project can be predicted according to Eq. 11:

11 $${m = }\frac{{{B}_{{\mathrm{initial}}} \times \Delta {B} \times {A}}}{{1000}},$$

where *A* is the restored area in *m*^2^. Rearranging Eq. 11, the size of the restored area required to offset any given loss is given by:

12 $${A} = \frac{{1000 \times {m}}}{{{B}_{{\rm{initial}}} \times \Delta {B}}}.$$

For example, if the initial biomass density was 9.68 g/m^2^ and the Δ*B* was 2.29, and the Bruce Generating Stations were required to offset ~2000 kg of fish annually, the area of restored habitat needed would be 90,223.34 m^2^, or 9.02 ha.

**Fig. 2:**
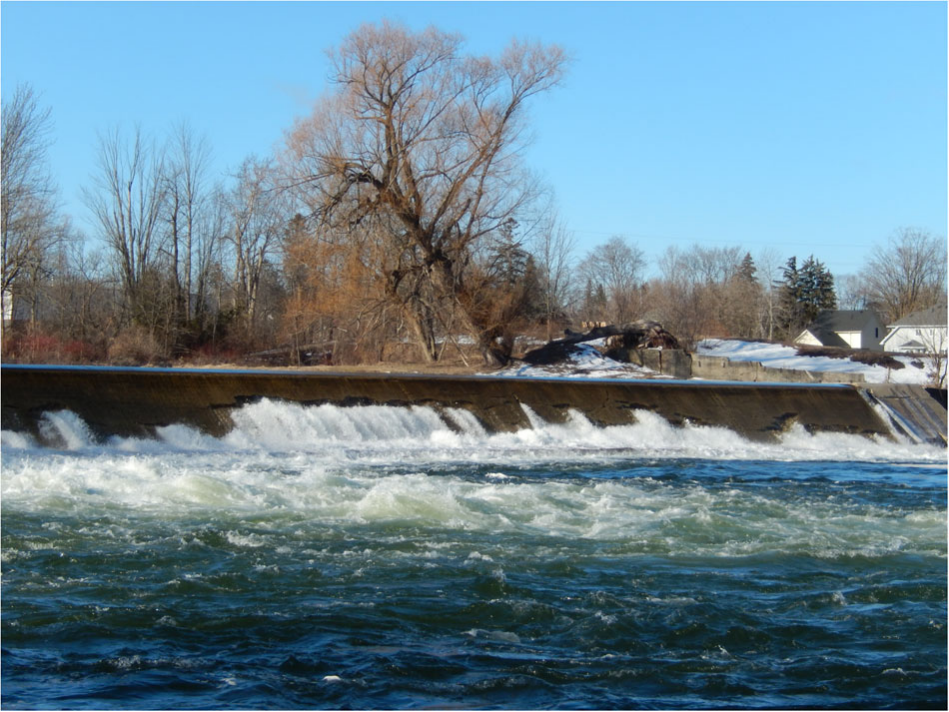
Truax Dam, located on the Saugeen River in Walkerton, ON, 50 km inland from Lake Huron. Removal of this dam is expected to increase fish production in the main stem river and upstream tributaries, offsetting losses due to impingement and entrainment at Bruce Station

A suite of projects in the vicinity of the Bruce Generating Stations has been identified and the projects have been ranked according to their over all potential to improve/create new fish habitat and increase fish productivity. The project that would provide the greatest potential offset would be removal of the Truax Dam on the Saugeen River (Walkerton, ON), ~50 km inland from Lake Huron (Fig. 2). This dam was built in 1919 and has long been identified as a major barrier to upstream passage for the entire fish community.

The Truax Dam Removal will offset I&E losses of Lake Huron fish species by increasing inland fish productivity in the Saugeen River watershed, which flows into Lake Huron. Some of the species that will benefit from the offset project move freely between lakes and rivers, so that increase production in the Saugeen River and its tributaries will lead directly to increased abundance of these species in Lake Huron. Examples include Brown Trout (*Salmo trutta*), Channel Catfish, Chinook Salmon (*Oncorhynchus Tshawytscha*), Coho Salmon (*Oncorhynchus kisutch*), Rainbow Trout (*Oncorhynchus mykiss*), and various sucker species.

**Fig. 3:**
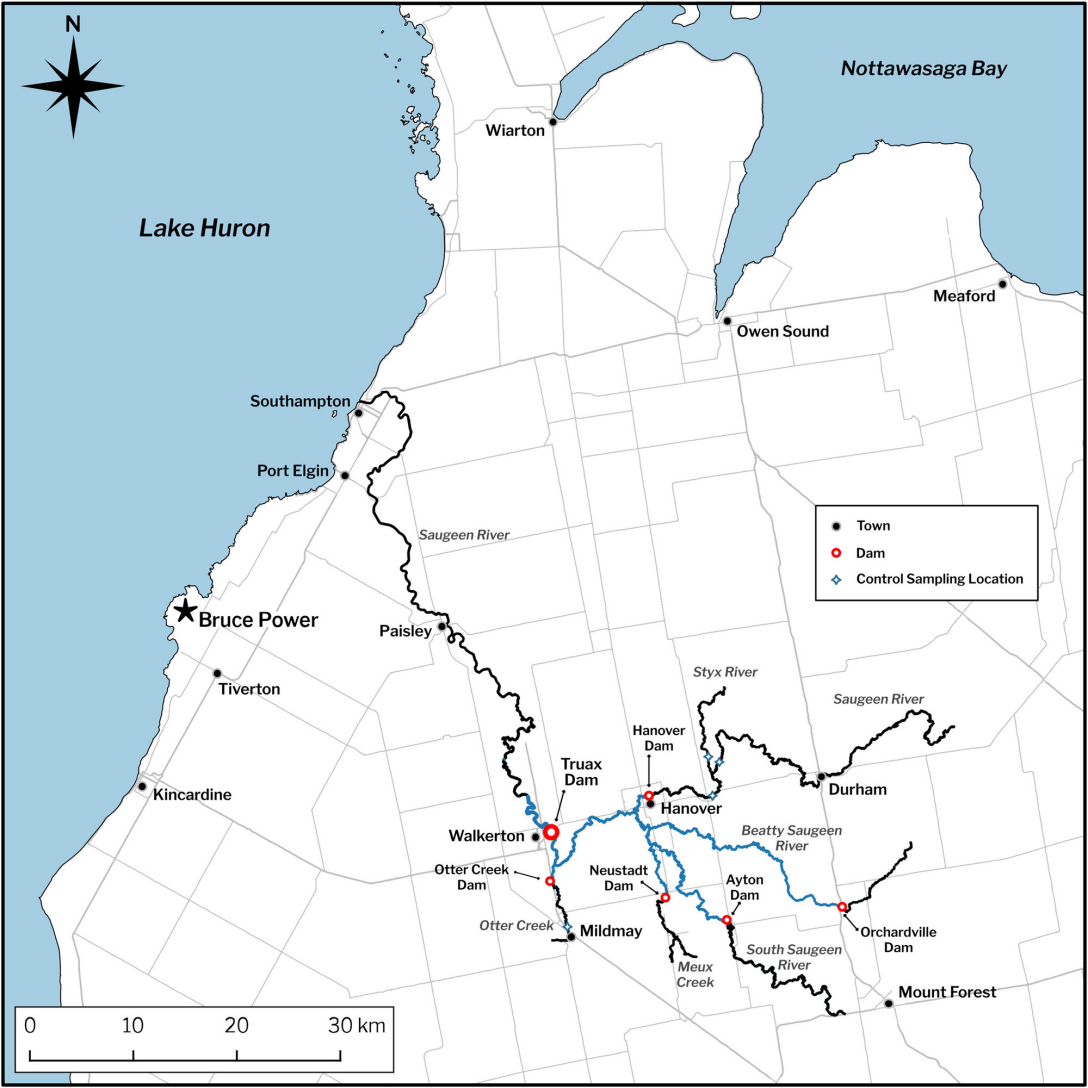
The Saugeen River watershed above Truax Dam

For preliminary planning purposes, the restored area is assumed to be equal to the length of the Saugeen River between the Truax Dam and the Maple Hill Dam (a 13.4 km stretch of river), including a small portion of Otter Creek (Fig. 3). Using GIS technology, the size of this area is estimated to be 94.3 ha, or ~10 times the area needed to replace I&E losses at the Bruce Generating Stations.

Although it appears from these calculations that removal of the Truax Dam should provide an increase in fish production far in excess of the amount required to offset I&E losses at the Bruce Generating Stations, the calculations were based on literature-derived estimates of initial biomass density (*B*_*initial*_) and fractional increase in biomass density (Δ*B*) due to dam removal. If the actual values of these parameters in the Saugeen River are lower than the values assumed above, then the actual increases in biomass density and productive capacity following dam removal will be lower than the estimate derived above. However, Eqs. 10 and 12 can be used to calculate the magnitude of increase in biomass density that would be required to achieve the necessary offset given the known size of the restoration area.

Rearranging Eq. 12,

13 $${B}_{{\rm{initial}}} \times \Delta {B} = \frac{{1000 \times {m}}}{{A}}.$$

Rearranging Eq. 10,

14 $${B}_{{\rm{initial}}} \times \Delta {B} = {B}_{{\rm{final}}} - {B}_{{\rm{initial}}}.$$

Therefore,

15 $${B}_{{\rm{final}}} - {B}_{{\rm{initial}}} = \frac{{1000 \times {m}}}{A}.$$

Given the known values of *m* and *A*, the incremental increase in biomass density averaged over the entire project area needed to offset the station losses would be 2.12 g/m^2^. This value is consistent with observed increases in biomass density in other dam removal projects (see Table 5), and is most likely conservative. Given that published *P*/*B* ratios for Canadian freshwater fish communities are in most cases greater than 1 (Randall et al. 2017), the community fish production associated with a 2.12 g/m^2^ increase in biomass density would likely be somewhat greater than 2.12 g/m^2^.

## Project Implementation

The calculations documented above demonstrate that it is technically feasible to offset losses in fish community productivity due to I&E by implementing habitat restoration projects that increase fish community productivity to an equal or greater extent. This demonstration is required for Bruce Power’s application for *Fisheries Act* authorization. However, they do not prove that the specific restoration project that has been proposed, i.e., removal of the Truax Dam, will provide the needed offset. In the long run, Bruce Power must demonstrate that the offset promised in the Application is actually being achieved.

A rigorous monitoring program that documents fish community production in the project area both before and after dam removal will be necessary. The detailed monitoring that is now underway is beyond the scope of this paper, however, a brief overview is provided here. The program is based on the Before-After-Control-Impact (BACI) design (Stewart-Oaten et al. 1986), which will permit distinguishing the effects of dam removal on fish communities from changes due to unrelated causes such as inter-annual variations in regional precipitation and stream runoff. To evaluate changes in fish biomass and production resulting from removal of the Truax Dam, physical habitat assessments and electrofishing surveys are being conducted at predetermined fixed sites along the main stem of the Saugeen River, established both upstream and downstream of the Truax Dam (near-field and far-field sites), as well as at two isolated control sites in the Saugeen River, upstream of the Hanover Dam near the community of Allan Park (Fig. 3). Biomass and production changes are monitored with electrofishing being conducted in several Saugeen River tributaries above Truax Dam. Consistent with the BACI design, tributary control sites have been established in Otter Creek in Mildmay, and upstream of Allan Park, in the Styx River.

All control sites are situated upstream of the impacted area and isolated from downstream areas by dams that do not permit upstream fish passage. In this regard they are isolated from the effects of the dam removal, but are located within the same watershed as the impact area. Hence, they are exposed to the same patterns of environmental variability and should host a similar complement of species.

Preremoval sampling provides site-specific estimates of initial biomass (*B*_*i*_) to replace the literature-derived value used in the preliminary calculations, and also will provide a baseline estimate of HPI. Postremoval sampling is currently scheduled for 2020–2023 and 2026 to measure the actual change in production resulting from the dam removal. If insufficient new production occurs, then Bruce Power will use Lake Trout stocking to fulfill the remaining offset that is needed until additional long-term offset projects can be implemented.

## Discussion

The goal of the Canadian *Fisheries Act* is to ensure that development activities do not reduce the productive capacity of fish habitat in Canadian waters. This goal is easy to state, but in practice often difficult to achieve. For Bruce Power, the *Fisheries Act* requires the facility owner to implement environmental restoration projects that replace the productive capacity of fish lost due to impingement and entrainment with new productive capacity provided through fish habitat restoration. However, the species susceptible to impingement and entrainment are different from the species that can be produced through feasible restoration projects, moreover, the data provided through monitoring of I&E at a power plant are not directly comparable to the data provided through field studies at restoration sites. Agency guidance provides no clearly applicable approaches that a power plant operator can rely on to balance losses and gains and demonstrate compliance with the act.

The approach documented in this paper draws on published scientific literature from a variety of sources to express I&E losses and restoration project gains in a common metric that can be used to scale the restoration projects needed to offset the losses. We have demonstrated the application of the approach to a specific restoration project, removal of the Truax Dam on the Saugeen River, Ontario.

One additional conceptual issue should be at least briefly noted: whether it is acceptable under the *Fisheries Act* to offset impacts on one type of fish habitat with restoration of another type of habitat. The Bruce Generating Stations impinge and entrain fish that inhabit Lake Huron, but the proposed restoration project involves removal of a dam 50 km inland from the lake. This offset is consistent with the Fisheries Productivity Investment Policy, as the Lake Huron ecosystem includes the associated watersheds. Many important commercial and recreational fish species utilize tributaries such as the Saugeen River as spawning and nursery habitat. Moreover, forage species produced in the river can enter the lake and be consumed by lake-dwelling predators. Therefore, increasing the productive capacity of the Saugeen River also increases the productive capacity of eastern Lake Huron, more than offsetting the loss of fish caused by the operation of the Bruce Generating Stations.

### Supplementary information

Supplementary Information

## Appendix

**Development of Adjusted EA Model**

The following modification of the EA model to account for impingement of older fish is based on the theory of reproductive value (Fisher 1930; Gotelli 2008). Reproductive value is defined as the relative number of offspring that remain to be born to an individual of a given age. The reproductive value approach can be used to calculate the expected future egg production of a fish older than age-1 compared to a 1-year-old fish. This calculation provides a weighting factor that can be used to convert numbers of fish impinged at ages greater than 1 to numbers of age-1 fish with an equivalent reproductive value.

The following equation expresses the expected lifetime egg production of an age-1 female fish:

16 $$\widehat E_{L1} = \mathop {\sum}\limits_{i = 1}^{A_{\rm{max}}} {S_{1i}M_iE_i}.$$

$\widehat E_{L1}$ = expected lifetime egg production of an age-1 female fish. *S*_*1i*_ = probability of survival from age-1 to age *i*. *M*_*i*_ = fraction of age *i* females that are sexually mature. *E*_*i*_ = average number of eggs produced per mature age *i* female. *A*_max_ = oldest age present in the population

The remaining lifetime egg production of a female fish impinged at age *x* (*x* > 1) is given by:

17 $$\widehat E_{Lx} = \mathop {\sum}\limits_{i = x}^{A_{\rm{max}}} {S_{xi}M_iE_i},$$

*S*_*xi*_ = probability of survival from age *x* to age *i.*

The ratio of expected lifetime egg production at age *x* to lifetime egg production at age-1 expresses the reproductive value of an age *x* fish relative to an age-1 fish:

18 $$W_{x1} = \frac{{E_{Lx}}}{{E_{L1}}}.$$

It could be argued that in calculating the weighting factor discussed here expected lifetime egg production should account for the proportion of fish in the population that are female. This would require adjusting Eqs 16 and 17 to include an additional term *F*_*i*_, the fraction of fish at a given age that is female. However, as long as males and females of a given age are equally susceptible to impingement, the *F*_*i*_ terms would cancel out of Eq 18.

It follows that:

19 $${\rm{NEQ}}_{1} = N_{x}W_{x1}.$$

*N*_*x*_ = number of fish impinged at age *x*, *W*_*x1*_ = weighting factor for age *x* fish, *NEQ*_*1*_ = adjusted number of age-1 equivalents for fish impinged at age *x*

It should be noted that Equation 19 is also applicable to eggs, larvae, and juvenile fish less than one year of age. For these fish, the expected lifetime egg production (*E*_*Lx*_) is equal to the expected lifetime egg production of an age-1 fish multiplied by the fraction of stage *x* fish expected to survive to reach age-1. Therefore, the weighting factor is calculated as:

20 $$W_{x1} = \frac{{E_{L1}S_{x,1}}}{{E_{L1}}} = S_{x,1}.$$

Thus, for all fish less than 1 year of age, the standard EA model already correctly accounts for the relative reproductive value of these fish compared to age-1 fish.

21 $${\rm{NEQ}}_{1} = S_{x,1}N_x$$

Reproductive value theory also underlies the Spawning Potential Ratio (SPR) approach to fish stock assessment (Goodyear 1993; Rosenberg et al. 1994). In SPR analysis, the lifetime potential egg production of a one-year-old female fish is defined in exactly the same way as in EA analysis, using Eq. 16.

Harvesting decreases the probability of survival from one age to the next, and therefore decreases potential egg production. The SPR associated with a given fishery management policy is defined by:

22 $${\rm{SPR}} = \frac{{EL_{\rm{1fished}}}}{{EL_{\rm{1unfished}}}}$$

Assuming that fecundity is proportional to body mass, SPR can also be calculated using the expected biomass of a fish at each age as a surrogate for expected egg production.

Impinging a fish at age *x* (*x* > 1) is equivalent, from a reproduction perspective, to harvesting that fish. Hence, the weighting procedure documented here for converting impingement counts of fish older than age-1 to age-1 equivalents is fully consistent with the SPR methodology.

**Application to Lake Whitefish impinged at Bruce A and Bruce B Nuclear Generating Stations**

Using the life history parameters in Table 1, the weighting factors for 2–12-year-old Lake Whitefish derived using equations 16 through A-3 are listed in Table 6. With the adjustment for reproductive value, the 28 age-12 Lake Whitefish impinged equate to 363 age-1 equivalents. These 363 fish have the same reproductive value as the 28 age-12 Lake Whitefish lost due to impingement. Expressed as age-1 equivalent biomass, these 363 fish equate to 27.2 kg of age-1 Lake Whitefish. Table 6 also presents the numbers of Lake Whitefish of all ages impinged during 2013. The 73 Lake Whitefish impinged during 2013 collectively equate to 515 age-1 equivalent fish or 38.5 kg of age-1 equivalent biomass. These results imply that production of an additional 515 age-1 Lake Whitefish would be sufficient to offset the loss in reproductive value to the population of all of the Lake Whitefish impinged in 2013. For comparison, Table 6 also provides estimates of age-1 equivalent losses expressed as numbers of individuals and total age-1 biomass calculated using the unadjusted EA model (Eqs. 1–3). As discussed in the text, the values for fish age 2 and older represent fish that were harvested or died of natural causes between age-1 and the age of impingement; these fish were never exposed to the intake structure and do not provide a meaningful basis for calculating offse requirements.

**Table 6:** Calculation of age-1 equivalent lake whitefish losses using the reproductive value weighting method

		Reproductive value-weighted model			Unadjusted equivalent adult model		
Life stage	Number impinged	Age-specific Weighting factor	Annual Loss as Age-1 Equivalents	Annual age-1 equivalent biomass loss (kg)	Age -specific Weighting Factor	Annual loss as Age-1 Equivalents	Annual age-1 equivalent biomass loss (kg)
Juveniles	12	N/A	1	0.1	0.0	0	0
1	9	1.0	12^a^	0.9	1.3	12	1
2	10	1.9	25	1.8	2.0	20	2
3	4	3.0	15	1.2	3.2	13	1
4	4	4.7	27	2.1	5.6	22	2
5	2	6.2	21	1.6	12.8	26	2
6	2	6.9	24	1.8	31.4	63	5
7	2	7.3	25	1.9	77.3	155	12
8	0	7.7	0	0.0	190.2	0	0
9	0	7.8	0	0.0	467.7	0	0
10	0	7.9	0	0.0	1150.4	0	0
11	0	7.8	0	0.0	2829.6	0	0
12	28	7.5	363	27.2	6959.8	194,874	14,569
13	0	6.6	0	0.0	17,118.3	0	0
14	0	4.2	0	0.0	42,104.3	0	0
Total			515	38.5		195,184	14,592

Impingement counts are from the two Bruce Generating Stations combined for the year 2013. Calculations performed using the unadjusted age-1 equivalent model are provided for comparison. Life history parameters used in these calculations are listed in Table 1

^a^Age-1 equivalent losses of 1-year-old fish are greater than the number impinged due to application of the adjusted survival factor (Eq. 3), which assumes that these fish were impinged randomly during the 1-year interval between age 1 and age 2

## Footnotes

1 Lake Whitefish are assumed to be become large enough to be recruited into the commercial fishery at approximately age-4, and to be fully recruited at ages 5 and older.

## References

[CR1] Abdel-Fatah S, Tymoshuk J, Doka SE, Minns CK (2017) Habitat/ecosystem assessment tool (HEAT) guidance document (Draft). Central and Arctic Region, Fisheries and Oceans Canada.

[CR2] Barrell JP, Wong MC, Grant J (2014) Evaluating coastal habitat value through metrics of ecosystem function for use in habitat restoration. Canadian Technical Report of Fisheries and Aquatic Sciences, 3095, Fisheries and Oceans Canada

[CR3] Bowlby JN, Roff JC (1986). Trout biomass and habitat relationships in Southern Ontario Streams. Trans Am Fish Soc.

[CR4] Chapman DW, Gerking SD (1978). Production in fish populations. Ch. 1. Ecology of freshwater fish production.

[CR5] Clarke, KD, Bradford MJ (2014) A review of equivalency in offsetting policies. Canadian Science Advisory Secretariat, Fisheries and Oceans Canada, CSAS no. 2014/109

[CR6] de Kerckhove DT, Smokorowski KE, Randall RG (2008) A primer on fish habitat models. Canadian Technical Report of Fisheries and Aquatic Sciences 2817

[CR7] Fisheries and Oceans Canada (1991) Guidelines for minimizing entrainment and impingement of aquatic organisms at marine intakes in British Columbia. Canadian Manuscript Report of Fisheries and Aquatic Sciences No. 2098

[CR8] Ebener MP, Brenden TO, Jones ML (2010). Estimates of fishing and natural mortality rates for four lake whitefish stocks in northern lakes Huron and Michigan. J Gt Lakes Res.

[CR9] Electric Power Research Institute (EPRI) (2004) Extrapolating impingement and entrainment losses to equivalent adults and production foregone. Report no. 1008471. Electric Power Research Institute, Palo Alto, CA

[CR10] EPRI (2012). Comprehensive update of fish life history parameter values. Report no. 1023103.

[CR11] Fisheries and Oceans Canada (2013a) Fisheries Act. R.S.C., 1985, C. F-14. Last amended on April 5, 2016

[CR12] Fisheries and Oceans Canada (2013b) Fisheries protection policy statement. DFO/13-1904

[CR13] Fisheries and Oceans Canada (2013c) Fisheries productivity investment policy: a proponent’s guide to offsetting. DFO/13-1905

[CR14] Fisheries and Oceans Canada (2014) Science advice on offsetting techniques for managing the productivity of freshwater fisheries. Canadian Science Advisory Secretariat Science Advisory Report 2013/074

[CR15] Fisher RA (1930). The genetical theory of natural selection.

[CR16] Goodyear CP (1993) Spawning stock biomass per recruit in fisheries management: foundation and current use. In: Smith SJ, Hunt JJ, Rivard D (eds) Risk evaluation and biological reference points for fisheries management. Canadian Special Pubilication in Fisheries and Aquatic Sciences. 120, p 67–81

[CR17] Gotelli NJ (2008). A primer of ecology.

[CR18] Hogg RS, Coghlan SM, Zydlewski J, Gardner C (2015). Fish community response to a small-stream dam removal in a Maine Coastal River Tributary. Trans Am Fish Soc.

[CR19] Horst TJ, Saila SB (1975). The assessment of impact due to entrainment of ichthyoplankton. Fisheries and energy production: a symposium.

[CR20] Kornis MS, Weidel BC, Powers SM, Diebel MW, Cline TJ, Fox JM, Kitchell JF (2015). Fish community dynamics following dam removal in a fragmented agricultural stream. Aquat Sci.

[CR21] Minns CK, Randall RG, Smokorowski KE, Clarke KD, Vélez-Espino A, Gregory RS, Courtenay S, LeBlanc P (2011). Direct and indirect estimates of the productive capacity of fish habitat under Canada’s Policy for the Management of Fish Habitat: where have we been, where are we now, and where are we going?. Can J Fish Aquat Sci.

[CR22] Muir AM, Ebener MP, He JX, Johnson JE (2008). A comparison of the scale and otolith methods of age estimation for lake whitefish in Lake Huron. N Am J Fish Manag.

[CR23] Muir AM, Arts MT, Koops MA, Johnson TB, Krueger CC, Sutton TM (2014). Reproductive life-history strategies in lake whitefish (*Coregonus clupeaformis*) from the Laurentian Great Lakes. Can J Fish Aquat Sci.

[CR24] Neves RJ, Pardue GB (1983). Abundance and production of fishes in a small Appalachian stream. Trans Am Fish Soc.

[CR25] Portt CB, Balon EK, Noakes DLG (1986). Biomass and production of fishes in natural and channelized streams. Can J Fish Aquat Sci.

[CR26] Rago PJ (1984). Production foregone: an alternative method for assessing the consequences of fish entrainment and impingement losses at power plants and other water intakes. Ecol Model.

[CR27] Randall RG, Minns CK (2000). Use of fish production per unit biomass ratios for measuring the productive capacity of fish habitats. Can J Fish Aquat Sci.

[CR28] Randall RG, Minns CK (2002). Comparison of a Habitat Productivity Index (HPI) and an Index of Biotic Integrity (IBI) for measuring the productive capacity of fish habitat in nearshore areas of the Great Lakes. J Gt Lakes Res.

[CR29] Randall, R, Cunjak R, Gibson J, Reid S, and Velez-Espino A (2014) Using electrofishing data to determine regional bencmarks of habitat productive capacity. Canadian Science Advisory Secretariat (CSAS) Research Document 2013/095

[CR30] Randall, RG, Bradford MJ, de Kerckhove DT, van der Lee A (2017) Determining regional benchmarks of fish productivity using existing electrofishing data from rivers: proof of concept. Canadian Science Advisory Secretariat (CSAS) Research Document 2017/018

[CR31] Ricker WE (1946). Production and utilization of fish populations. Ecol Monogr.

[CR32] Rosenberg A, Mace P, Thompson G, Darcy G, Clark W, Collie J, Gabriel W, MacCall A, Methot R, Powers J, Restrepo V, Wainwright T, Botsford L, Hoenig J, Stokes. K (1994). Scientific review of definitions of over-fishing in U.S. fishery management plans. NOAA Technical Memorandum NMFS/F-SPO-17.

[CR33] Stewart-Oaten A, Murdoch WW, Parker KR (1986). Environmental impact assessment: pseudoreplication in time?. Ecology.

[CR34] U.S. Environmental Protection Agency (USEPA) (1977) Guidance for evaluating the adverse impact of cooling water intake structures on the aquatic environment (draft): Section 316(b), P.L. 92-500. Office of Water Enforcement, Permits Division, Industrial Permits Branch, Washington, D.C

[CR35] USEPA (2002). Case study analysis for the proposed Section 316(b) existing facilities rule. EPA 821-R-02-002.

[CR36] USEPA (2014). Benefits analysis for the final Section 316(b) existing facilities rule. EPA-821-R-14-005.

[CR37] Wang H-Y, HööK TO, Ebener MP, Mohr LC, Schneeberger PJ (2008). Spatial and temporal variation of maturation schedules of lake whitefish (*Coregonus clupeaformis*) in the Great Lakes. Can J Fish Aquat Sci.

