# Supplementary material for: Quantifying Restoration Offsets at a Nuclear Power Plant in Canada
Source: Environ Manage. 2019 Oct 19;64(5):593–607. doi: 10.1007/s00267-019-01214-2 (PMC6838036; doi:10.1007/s00267-019-01214-2)
Supplement: Supplementary file 1 — Supplementary Information [file 267_2019_1214_MOESM1_ESM.docx]

Table A-1. Calculation of age-1 equivalent Lake Whitefish losses using the reproductive value weighting method. Impingement counts are from the two Bruce Generating Stations combined for the year 2013. Calculations performed using the unadjusted age-1 equivalent model are provided for comparison. Life history parameters used in these calculations are listed in Table 1.

|  |  | Reproductive value-weighted model | | | Unadjusted equivalent adult model | | |
| --- | --- | --- | --- | --- | --- | --- | --- |
| Life stage | Number impinged | Age-specific Weighting factor | Annual Loss as Age-1 Equivalents | Annual age-1 equivalent biomass loss (kg) | Age -specific Weighting Factor | Annual loss as Age-1 Equivalents | Annual age-1 equivalent biomass loss (kg) |
| juveniles | 12 | N/A | 1 | 0.1 | 0.0 | 0 | 0 |
| 1 | 9 | 1.0 | 12^a^ | 0.9 | 1.3 | 12 | 1 |
| 2 | 10 | 1.9 | 25 | 1.8 | 2.0 | 20 | 2 |
| 3 | 4 | 3.0 | 15 | 1.2 | 3.2 | 13 | 1 |
| 4 | 4 | 4.7 | 27 | 2.1 | 5.6 | 22 | 2 |
| 5 | 2 | 6.2 | 21 | 1.6 | 12.8 | 26 | 2 |
| 6 | 2 | 6.9 | 24 | 1.8 | 31.4 | 63 | 5 |
| 7 | 2 | 7.3 | 25 | 1.9 | 77.3 | 155 | 12 |
| 8 | 0 | 7.7 | 0 | 0.0 | 190.2 | 0 | 0 |
| 9 | 0 | 7.8 | 0 | 0.0 | 467.7 | 0 | 0 |
| 10 | 0 | 7.9 | 0 | 0.0 | 1150.4 | 0 | 0 |
| 11 | 0 | 7.8 | 0 | 0.0 | 2829.6 | 0 | 0 |
| 12 | 28 | 7.5 | 363 | 27.2 | 6959.8 | 194874 | 14569 |
| 13 | 0 | 6.6 | 0 | 0.0 | 17118.3 | 0 | 0 |
| 14 | 0 | 4.2 | 0 | 0.0 | 42104.3 | 0 | 0 |
| **Total** |  |  | **515** | **38.5** |  | **195184** | **14592** |

^a^Age-1 equivalent losses of one-year-old fish are greater than the number impinged due to application of the adjusted survival factor (Equation 3), which assumes that these fish were impinged randomly during the 1-year interval between age 1 and age 2
